# Longitudinal monitoring of SARS-CoV-2 viral load in self-collected saliva from health care workers during breakthrough infections to spare working days

**DOI:** 10.1128/spectrum.02555-23

**Published:** 2023-11-20

**Authors:** Valeria Caneparo, Carmela Rinaldi, Daniela Ferrante, Paolo Ravanini, Irene Lo Cigno, Silvia Cavalieri, Marisa Gariglio, Cinzia Borgogna

**Affiliations:** 1 CAAD-Center for Translational Research on Autoimmune and Allergic Disease, University of Piemonte Orientale, Novara, Italy; 2 Aging Project, Department of Translational Medicine, University of Piemonte Orientale, Novara, Italy; 3 Education and Research area, Health Professions Direction, Maggiore Della Carità Hospital, Novara, Italy; 4 Medical Statistics, Department of Translational Medicine, University of Piemonte Orientale, Novara, Italy; 5 Unit of Microbiology and Virology, Department of Laboratory Medicine, "Maggiore della Carità" Hospital, Novara, Italy; 6 Virology Unit, Department of Translational Medicine, University of Piemonte Orientale, Novara, Italy; 7 Occupational Health Unit, Specialist Medical and Oncological Department, "Maggiore della Carità" University - Hospital, Novara, Italy; University of Mississippi Medical Center, Jackson, Mississippi, USA

**Keywords:** SARS-CoV-2, droplet digital PCR, viral load, health care workers

## Abstract

In this prospective study, we used droplet digital (dd)PCR to determine the SARS-CoV-2 viral load determination in saliva samples collected from two cohorts of Italian health care workers (HCWs) who had tested positive for SARS-CoV-2 breakthrough infections in nasopharyngeal swabs (NPS). The study encompasses two distinct periods: one from February to May 2021 (*n* = 16), characterized by Alpha variant predominance, and another from January to March 2022 (*n* = 23), marked by the prevalence of the Omicron variant. To assess the temporal pattern of virus shedding, we performed dd-PCR analysis of self-collected longitudinal saliva harvested the day after NPS testing and every 3 days until SARS-CoV-2 negativization, determined by real-time PCR (RT-PCR). Using saliva harvested within 24 h from the initial SARS-CoV-2-positive NPS tested by real-time quantitative PCR (RT-qPCR), we found that 11/16 [68.7%, confidence interval (CI) 95% 44.0%–93.4%] HCWs in the “Alpha wave” and 6/23 (26.1%, CI 95% 7.1%–45.1%) in the “Omicron wave” were negative for the presence of SARS-CoV-2 by ddPCR. The viral loads obtained with these samples were negatively correlated with the cycle threshold (Ct) values measured in the NPS specimens (*P* < 0.0001). The median Ct values of the double-positive (RT-qPCR and ddPCR) subjects were significantly higher when compared to the single-positive (RT-qPCR only) ones. In addition, we found that using ddPCR in self-collected saliva, 80% of the HCWs in the “Alpha wave” and 53% in the “Omicron wave” would have been freed from isolation within four working days from the initial positive NPS, sparing 176 working days among the HCWs enrolled in the study. Overall, this study highlights the effectiveness of using ddPCR in self-collected saliva as part of a surveillance program. It ultimately aims to identify individuals who still test positive by RT-PCR in NPS but retains a residual, non-active infection. This diagnostic approach is likely to not only prevent unnecessary isolation but also spare working days.

**IMPORTANCE:**

Real-time quantitative PCR (RT-qPCR) on nasopharyngeal swabs (NPS) has been used as the standard method for detecting and monitoring SARS-CoV-2 infection during the pandemic. However, NPS collection often causes discomfort and poses a higher risk of transmission to health care workers (HCW). Furthermore, RT-qPCR only provides relative quantification and does not allow distinguishing those samples with residual, no longer active infection, whereas droplet digital PCR (ddPCR) allows for precise quantification of viral load, offering greater sensitivity and reproducibility. This study highlights the effectiveness of using self-collected saliva as a convenient and reliable sampling method. By utilizing ddPCR to measure the SARS-CoV-2 viral load in saliva samples, individuals with low or undetectable viral loads can be quickly identified. This approach is particularly advantageous for surveillance programs targeting HCW, as it enables the early identification and release of uninfected personnel, minimizing lost workdays. Additionally, analyzing viral load in saliva samples by ddPCR is valuable in determining virus shedding duration across different SARS-CoV-2 variants, informing transmission and disease control. Finally, testing saliva could overcome the detection of historic cases due to prolonged RNA swabbing past-infection and the unnecessary exclusion of those individuals from the workplace.

## INTRODUCTION

Viral load and shedding kinetics are crucial determinants of viral transmission as they influence the duration of the infectiousness, a critical parameter to inform effective control measures and disease modeling. The acquisition of these parameters, despite being quite challenging, has gained particular importance in the context of the current SARS-CoV-2 pandemic as it has helped optimize testing strategies for emerging variants of concern (VOCs), ultimately improving our preparedness to future viral pandemics. Thus, identifying the factors that influence infectious viral shedding and estimating the period during which SARS-CoV-2-infected subjects are contagious are key to guiding public health interventions and limiting viral transmission (1, 2).

Saliva has been shown to be an accurate diagnostic tool for the diagnosis of SARS-CoV-2 infection, which not only might lower the chances of detecting past infections but also reduces patient discomfort and the risk of transmission among health care workers (HCWs) during sample collection as compared to nasopharyngeal swabs (NPS) (3 – 6). Moreover, saliva seems to be more sensitive early in the infection, and high viral loads in this biological fluid have been associated with increased transmissibility, primarily due to the presence of viral particles in the saliva droplets of infected individuals (7 – 11). However, direct comparisons of viral load values between saliva specimens and NPS have led to controversial data as they were found to be equivalent in some cases but different in others (3 – 6, 11, 12). Although these discrepancies may be ascribable to differences in disease status (e.g., symptomatic vs asymptomatic COVID-19), they may also be due to the different techniques used to assess the viral load—e.g., standard real-time quantitative PCR (RT-qPCR) using the Ct values as a proxy or droplet digital PCR (ddPCR). In particular, the ddPCR method, which allows the absolute quantification of nucleotide sequences, appears to have a higher analytical sensitivity and better reproducibility than qPCR (13 – 15).

During the ongoing SARS-CoV-2 pandemic, multiple variants of concern (VOCs) have emerged (16). The B.1.1.7 or Alpha variant was shown to have a much higher transmissibility than other variants of no concern, rapidly becoming the dominant circulating VOC in most countries, including Italy, in the period from March to June 2021. Since January 2022, the highly transmissible B.1.1.529 or Omicron variant has become the most prevalent SARS-CoV-2 variant worldwide, including Italy (17).

Knowledge of virus dynamics is essential for devising strategies to achieve epidemiological control, especially in medical/hospital settings where identifying infectious HCWs is crucial to prevent the spread of the virus among co-workers and patients and to maintain workforce capacity (1, 18).

In this prospective study, we used viral load determination by ddPCR in self-collected saliva samples to assess the temporal profiling of virus shedding in a cohort of regularly screened HCWs who tested positive for SARS-CoV-2 breakthrough infections. We show that self-collected saliva is a reliable and patient-friendly sampling method for the determination of the SARS-CoV-2 viral load by ddPCR that allows us to quickly identifying those subjects who display low or undetectable viral loads. In the context of HCW surveillance programs, our findings highlight the need to perform second-level testing, such as viral load analysis by ddPCR, to release uninfected workers earlier, thereby sparing working days.

## MATERIALS AND METHODS

### Patients, samples, and data collection

During the study period, HCWs adhered to the surveillance protocol implemented by the “Maggiore della Carità” University Hospital in Novara, irrespective of their exposure or presence of symptoms. The screening test frequency varied, occurring either weekly or monthly, contingent upon the specific department guidelines. This prospective single-center diagnostic study was conducted among HCWs who had willingly provided written informed consent. Participants had a laboratory-confirmed SARS-CoV-2 infection identified by reverse transcription quantitative PCR on NPS during two study periods: February to May 2021 and January to March 2022, when the Alpha and Omicron variants, respectively, were the main circulating SARS-CoV-2 variants in Italy. Biological samples were provided by the UPO Biobank after scientific and ethical review and approval (CE13/21; UPOBB_2021_06_AOU-ddPCR) by the Ethics Committee of Maggiore della Carità Hospital. Samples and associated data were pseudonymized and recorded on the REDCap web application in compliance with current EU general data protection regulation (GDPR) and Italian legislation on the protection of sensitive data and privacy. Data for SARS-CoV-2-infected HCWs were collected from clinical records and through telephonic interviews.

### SARS-CoV-2 RNA detection in nasopharyngeal swabs

NPS were collected by trained HCWs in tubes containing 2 mL of 1 × Hanks’ balanced salt solution without phenol red (Thermo Fisher Scientific, Waltham, MA, USA). Commercially available kits were used to detect SARS-CoV-2 virus [Allplex 2019-nCoV Assay (Seegene, Seoul, Republic of Korea): Xpert Xpress CoV-2 plus (GeneXpert, Cepheid, Sunnyvale, CA, USA); Simplexa COVID-19 Direct (DiaSorin Molecular, Cypress, CA, USA); Viasure SARS-CoV-2 BD MAX System (CERTest Biotec, Zaragoza, Spain)]. NPS were processed according to the manufacturer’s instructions. A specimen was considered positive if one target gene had a Ct <40 (19).

### SARS-CoV-2 RNA quantification in saliva

Subjects were requested to harvest saliva the day after testing and every 3 days until they became SARS-CoV-2-negative by ddPCR. They were asked to self-collect an early morning saliva sample from the posterior oropharynx (i.e., coughed up by clearing the throat) before toothbrushing and breakfast.

Total RNA was extracted from 200 µL of saliva using the Maxwell 16 Viral Total Nucleic Acid Purification Kit (Promega, Madison, WI, USA) following the manufacturer’s instructions. SARS-CoV-2 genomic RNA was quantified using the QX200 Droplet Digital PCR System (ddPCR, Bio-Rad, Hercules, CA, USA) and the SARS-CoV-2 Droplet Digital PCR Kit (Bio-Rad, Hercules, CA, USA). SARS-CoV-2 quantification was expressed as a copy number/μL of saliva.

### Statistical analysis

Data were expressed as medians with interquartile ranges (IQR) or numbers with corresponding percentages, as appropriate. Continuous variables were compared using the Mann–Whitney test or Student’s *t*-test, according to the data distribution. Categorical variables were analyzed using the χ test or Fisher’s exact test. The relationships between saliva samples and Ct values were evaluated by computing the Spearman correlation coefficient. The Friedman test was used to evaluate the trend of measurements over time. All statistical tests were two-sided, and a *P*-value  <0.05 indicated statistical significance. Statistical analyses were performed using Stata 17 (StataCorp LLC, College Station, TX, USA). Receiver operating curve (ROC) analysis was used to evaluate the discrimination power of Ct values for viral load.

## RESULTS

**Fig 1:**
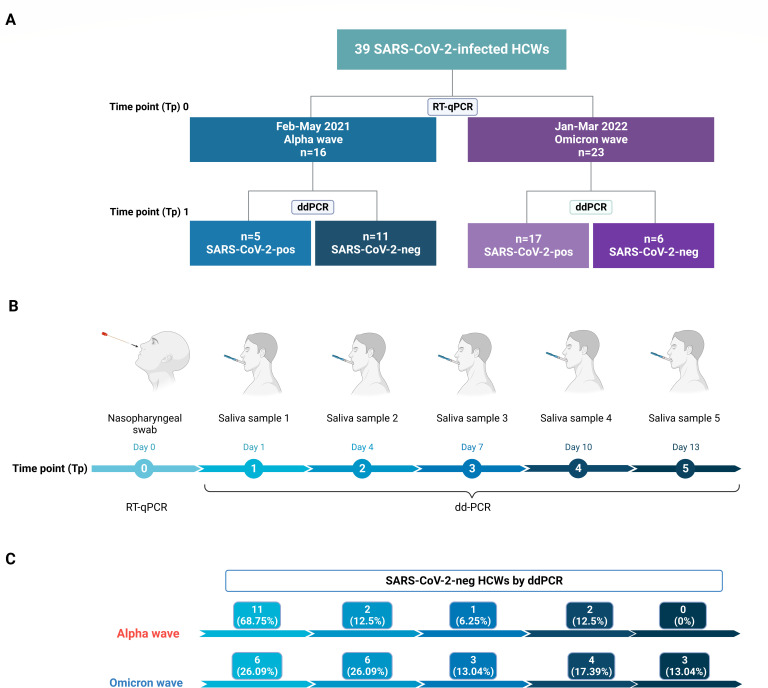
Study design. (**A**) Flowchart of the HCWs enrolled in the study. (**B**) Timeline of SARS-CoV-2 testing (RT-qPCR in NPS and ddPCR in saliva samples). HCWs who initially tested negative for saliva at time point one were subsequently sampled at later time points, and consistently, these subsequent samples yielded negative results. (**C**) Number and percentage of SARS-CoV-2-negative HCWs over time as determined by ddPCR analysis during the two study periods. This figure was created with Biorender.com.

During the two observation periods, February to May 2021 and January to March 2022, out of the 2,788 HCWs referring to our university-hospital in Novara (Northern Italy), 39 subjects in the first study period and 570 in the second one tested positive for SARS-CoV-2 infection by standard RT-qPCR of NPS. Among these, 39 patients signed the informed consent and provided us with self-collected saliva samples for longitudinal monitoring, as shown in Fig. 1A and B. In total, 144 saliva specimens were obtained from 39 subjects (at least three specimens from three different time points/subject). The patients only displayed mild symptoms at some stage or were asymptomatic, and none required hospitalization. The median age was 50 years (IQR 37–55), and the majority were women (*n* = 31; 79.5%). The clinical and demographic characteristics of the study patients are listed in Table 1. Regarding their occupational status, 18 subjects (46.1%) were nursing staff members, 6 (15.3%) administrative workers, 8 (20.5%) allied health professionals, and 7 (17.9%) physicians. When the subjects from the two study periods were stratified according to the presence of morbidities, the highest rates of subjects with 1 (6; 37.5%) or ≥2 morbidities (3; 18.8%) were found in the first study period (*P* = 0.02). These were as follows: Hashimoto’s thyroiditis in two cases, hypertension in three cases, autoimmune thyroiditis in one case, Sjögren syndrome in one case, psoriatic arthritis in one case, glaucoma in one case, arthrosis in one case, and asthma in three cases. Notably, most of these morbidities were associated with the presence of an immunity disorder, a risk factor for developing breakthrough infections, especially during the period of Alpha prevalence. In contrast, during the outbreak of the highly infectious Omicron variant, the presence of morbidities had no impact on the occurrence of breakthrough infections. Overall, 10 subjects had a prior SARS-CoV-2 infection that occurred from March to December 2020. The median interval from the last vaccine dose to SARS-CoV-2 detection was 57 days (IQR 41–74). During the first study period, four subjects did not receive any vaccine because they had become SARS-CoV-2 positive before their scheduled vaccination, while the remaining 12 subjects received two doses. In the second period, they were all vaccinated with two doses, and 22 out of 23 subjects received the booster dose.

**TABLE 1:** Characteristics of the SARS-CoV-2-infected health care workers (HCWs) enrolled in the study

	Total	Feb–May 2021	Jan–Mar 2022	
	N (%)	N (%)	N (%)	*P*-value
Age, median (IQR) years	50 (37–55)	50 (43–53)	41 (33–55)	0.47
Gender				
Male	8 (20.5)	5 (31.2)	3 (13.0)	
Female	31 (79.5)	11 (68.8)	20 (87.0)	0.23
Presence of comorbidities				
None	26 (66.7)	7 (43.7)	19 (82.6)	
1	10 (25.6)	6 (37.5)	4 (17.4)	
≥ 2	3 (7.7)	3 (18.8)	0 (0.0)	**0.02^*b*^**
Prior SARS-CoV-2 infection				
No	29 (74.4)	11 (68.7)	18 (78.3)	
Yes	10 (25.6)	5 (31.3)	5 (21.7)	0.71
Vaccine regimen				
No	4 (10.3)	4 (25.0)	0 (0.0)	
Yes (two doses)	13 (33.3)	12 (75.0)	1 (4.3)	
Yes (three doses)	22 (56.4)	0 (0.0)	22 (95.7)	

	Value (IQR)	Value (IQR)	Value (IQR)	*P*-value
Median viral load Tp1^*a*^ (copies/μL) (*n* = 22, 5, 17)	692.5 (155.6–4,680)	299.5 (236–323.5)	1335 (155.56–4,680)	0.319
Median Ct value Tp0 (*n* = 37, 16, 21)	23 (20–33)	34 (21–38)	21 (20–24)	**0.006**
Median Ct value Tp0 in SARS-CoV-2-neg by ddPCR (*n* = 16, 11, 5)	34 (25–37)	35 (31–39)	26 (24–29)	0.081
Median Ct value Tp0 in SARS-CoV-2-pos by ddPCR (*n* = 21, 5, 16)	20 (19–22)	20 (20–22)	20 (19–22)	>0.999

^*a*^ Tp1, Time point 1, and Tp0, Time point 0, as indicated in Fig. 1B.

^*b*^ Boldface indicates statistically significant results (P ≤ 0.05).

**Fig 2:**
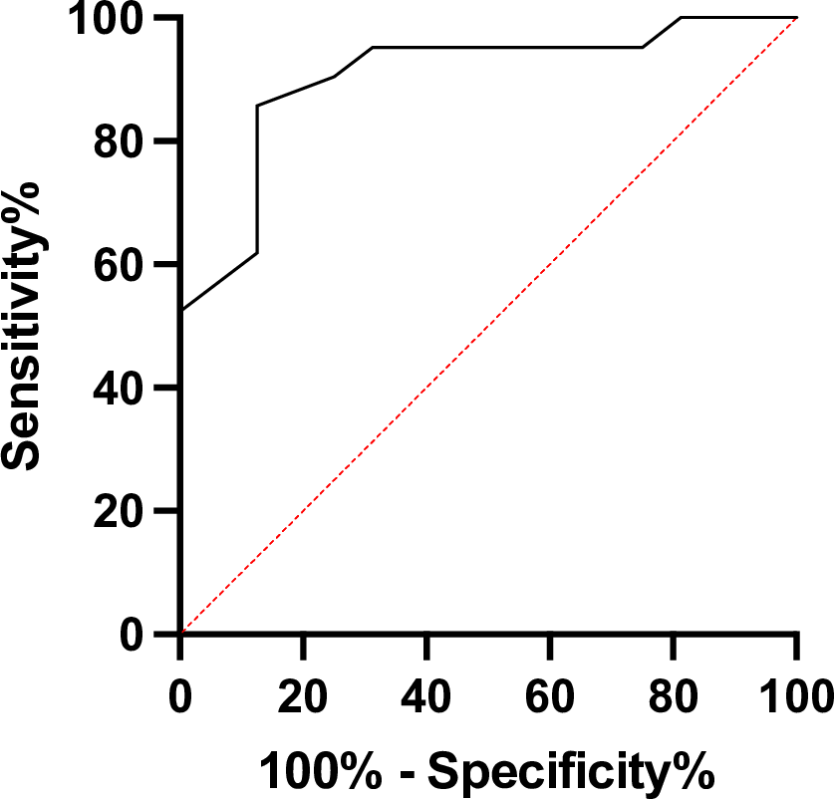
ROC curve. ROC curve analysis to evaluate the discriminating power of Ct values for viral load. (ROC AUC = 0.5, red line).

Using the self-collected saliva sample harvested at time point 1 (Fig. 1B), within 24 h from the SARS-CoV-2-positive NPS sampling, 11/16 (68.7%, CI 95% 44.0%–93.4%) HCWs in the “Alpha wave” and 6/23 (26.1%, CI 95% 7.1%–45.1%) in the “Omicron wave” turned out to be negative for the presence of the SARS-CoV-2 genome by ddPCR. Those subjects with a viral load detectable by ddPCR displayed a higher median value (1,335 copies/μL; IQR 155.56–4,680) in the second period than that of the first one (299.5 copies/μL; IQR 236–323.5) (Table 1). A possible explanation for this contentious data is that the higher viral loads observed in boostered vs vaccinated HCWs are more likely associated with the higher replication rate of the Omicron variant—whose outbreak occurred when HCWs were already boostered—compared to that of the Alpha variant (20, 21). However, this difference did not reach any statistical significance (*P* = 0.319). Consistently, the median Ct values of the RT-qPCR performed using the NPS harvested at time 0 were significantly higher in the “Alpha wave” group than those of the “Omicron wave” group (median Ct value 34, IQR 21–38 vs 21, IQR 20–24; *P* = 0.006, respectively). Notably, the viral load values observed at time point 1 were negatively correlated with the Ct values found at time point 0 (Spearman correlation r = −0.65, *P* < 0.0001). According to ROC curve analysis, the predictive power of the Ct values in the entire cohort was estimated as area under the ROC curve (AUC) 0.9048 (95% CI 0.80–1.00) (Fig. 2). Regarding the Ct values of those subjects negative for ddPCR, we found that the overall median Ct values in these patients were 34 (IQR 25–37), with values of 35 (IQR 31–39) and 26 (IQR 24–29) when the two subgroups were analyzed. As expected, the median Ct level in the double positive was steadily at 20, regardless of whether they were considered all together or divided into the two subgroups. However, when comparing the median Ct value of the double- vs single-positive subjects, we found statistically significant differences in both the entire cohort and the two subgroups (*P* < 0.0001). This confirms that, at the highest Ct values, the viral load in saliva can be extremely low or almost undetectable. Therefore, ddPCR analysis can be used to determine the potential infectivity status of individuals, as many reports have shown that subjects exhibiting low RNA levels in saliva (< 150 copies/μL) are unlikely to be infectious (22 – 24). This inference is crucial in making informed decisions regarding transmission risks and public health measures.

Notably, the exclusion criterion could not be established using a cut-off value of >34 Ct in NPS, which is usually regarded as a threshold for low viral load and improbable transmissible disease, as only 8/16 (50%) HCWs in the Alpha wave (excluding three negatives by ddPCR) and 1/23 (4.3%) in the Omicron wave (excluding five negatives by ddPCR) were above this cut-off value and would have been considered to have a low probability of transmission. Additionally, we observed that the Ct values were significantly lower in double-vaccinated individuals (median Ct value = 21.76) than in those who received booster shots (median Ct value = 23.14) individuals (*P* = 0.029). However, by performing a longitudinal analysis on 32 individuals over 14 days, we found no difference in the viral load trajectory between double-vaccinated and booster-shot recipients, indicating that booster immunizations lead to a reduction in detectable viral loads without significantly changing viral load dynamics over time.

**Fig 3:**
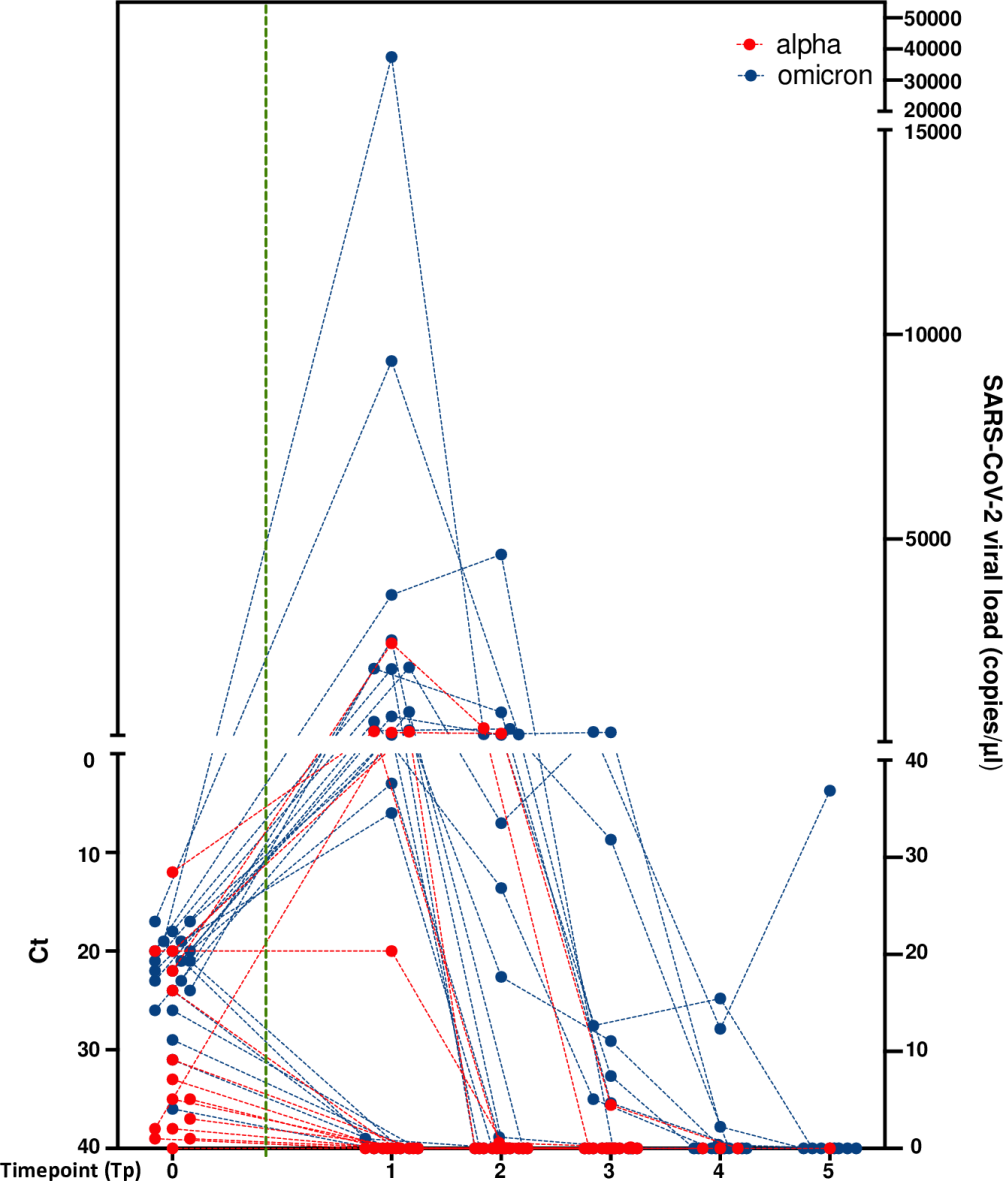
Longitudinal profiling of SARS-CoV-2 dynamics in HCWs. The figure displays RT-qPCR Ct values at time point 0 in NPS samples (left, y-axis: Ct values) and longitudinal profiling of SARS-CoV-2 viral load (copies/µl) as determined by ddPCR in saliva samples at time points 1 to 5 (right, y-axis: SARS-CoV-2 viral loads). Colored dots indicate the SARS-CoV-2 variants (Alpha in red; Omicron in blue). Dashed lines connect the different values (Ct and SARS-CoV-2 viral loads) at various time points on the same subject. Tp: Time point. The vertical green dotted line separates Ct values from viral load quantification.

As for the longitudinal assessment by ddPCR, we observed that viral load values in the saliva samples were significantly higher at the early time points and then gradually declined (Friedman test *P* = 0.0078) (Fig. 3). As shown in Fig. 1C and 3, during the first period, of the five positive subjects at Tp1, two became negative at Tp2, one at Tp3, and two at Tp4. The mean viral load values of the saliva samples were 242.2 copies/μL at Tp2 (three subjects) and 2.5 copies/μL at Tp3 (two subjects). During the second period, out of 17 SARS-CoV-2-positive HCWs at Tp1, 6 became negative at Tp2, 3 at Tp3, 4 at Tp4, and 3 at Tp5, while 1 remained positive at Tp5. The mean viral load of saliva samples was 1,355.1 copies/µL at Tp2 (11 subjects), 74.8 copies/μL at Tp3 (8 subjects), and 7.5 copies/µL at Tp4 (4 subjects).

### Conclusions

Our study has the following practical implications: first, SARS-CoV-2 viral load assessment in self-collected saliva by ddPCR can be used as a quick and easy approach to define infectious virus shedding and transmissibility in HCWs. Second, since infection with different SARS-CoV-2 VOCs in the two study periods resulted in highly distinct viral RNA loads over time, longitudinal monitoring of the viral load in self-collected saliva may help to quickly establish the duration of infectiousness of a given infectious agent or variant transmitted through the respiratory route. Finally, we show that relying on Ct values as a proxy of viral load, using a cut-off value of 34 (25), especially in a single sampling without a longitudinal assessment, significantly underestimates the number of non-infectious individuals. In addition, it is important to point out that the use of Ct values introduces an additional bias, given the availability of multiple real-time PCR (RT-PCR) assays that can potentially yield discordant results.

As part of the COVID-19 surveillance program during the timeframe of this study (February 2021 to March 2022), HCWs are required to be retested 10 days after an initial SARS-CoV-2-positive NPS test by RT-qPCR. However, the use of a sensitive technique like ddPCR would have allowed the HCWs enrolled in the study to return to work much earlier, saving a total of 176 working days. Specifically, during the first study period, when the Alpha variant was predominant, 80% of the subjects would have been released from isolation within four working days of the positive NPS. A similar trend, albeit to a lesser extent, would have also been observed during the second period of our study, characterized by the dominance of the fast-replicating Omicron variant, when 52% of SARS-CoV-2-positive HCWs would have been allowed to resume their duties within 4 days from the first positive test.

Several shortcomings of our study need to be listed, including the need to expand the number of subjects to enhance the statistical power of our analysis. In addition, we were unable to gather information about the transmissibility between close contacts, and the Ct values were obtained using RT-PCR kits from various manufacturers.

Altogether, our findings indicate that assessment of the viral load by ddPCR in self-collected saliva samples is an easy-to-do and highly sensitive assay that can be used to determine the duration of virus shedding among respiratory viruses and VOCs, which can be relevant for public health interventions. Indeed, virus isolation, the gold standard for determining the presence of infectious viruses in respiratory specimens, is time- and cost-consuming and requires specially trained personnel and biosafety level-3 laboratories, making detection of viable viruses through this protocol unsuitable for diagnostics and restricted to research. By contrast, estimating the viral load by ddPCR is affordable and does not require specialized infrastructure.

## Data Availability

The data supporting the views of this analysis are available from the corresponding author upon request.
